# Eptifibatide-Induced Severe Thrombocytopenia After ST-Elevation Myocardial Infarction (STEMI): A Case Report

**DOI:** 10.7759/cureus.29549

**Published:** 2022-09-25

**Authors:** Zaid Gheith, Areej Kilani, Tam Nguyen

**Affiliations:** 1 Internal Medicine, University of Texas Health Science Center at San Antonio, San Antonio, USA; 2 Internal Medicine, University of Jordan, Amman, JOR; 3 Cardiology, University of Texas Health Science Center at San Antonio, San Antonio, USA

**Keywords:** dapt, bleeding risk, drug-induced thrombocytopenia, thrombocytopenia, st-elevation myocardial infarction (stemi)

## Abstract

Eptifibatide is a platelet glycoprotein (GP) IIb/IIIa inhibitor that is used in certain cases of acute coronary syndrome, including those with high thrombus burden or with no-reflow. It can rarely be associated with severe thrombocytopenia, which brings up a dilemma in managing those patients who require antiplatelet therapy. We discuss a patient who had ST-elevation myocardial infarction (STEMI) and developed severe thrombocytopenia after eptifibatide infusion. He was managed with platelet transfusion, stopping eptifibatide, and interrupting dual antiplatelet therapy (DAPT).

## Introduction

ST-elevation myocardial infarction (STEMI) requires emergent revascularization and the need for percutaneous coronary intervention with a drug-eluting stent as a standard of care [1]. A subset of high-risk patients, including those with high thrombus burden or with no-reflow, might benefit from platelet glycoprotein (GP) IIb/IIIa inhibitors, putting them at a higher risk of bleeding [2]. We present a case of thrombocytopenia occurring within hours after eptifibatide infusion that was complicated by gross hematuria.

## Case presentation

A 69-year-old man with a past medical history of liver cirrhosis secondary to alcohol abuse presented to the emergency department with substernal non-radiating chest pain for two hours. It occurred when he was mowing the lawn and improved when he sat down. The pain was associated with fatigue and hand numbness. He had no associated shortness of breath or prior episodes of chest pain. He doesn’t smoke and quit drinking alcohol five years ago.

**Figure 1:**
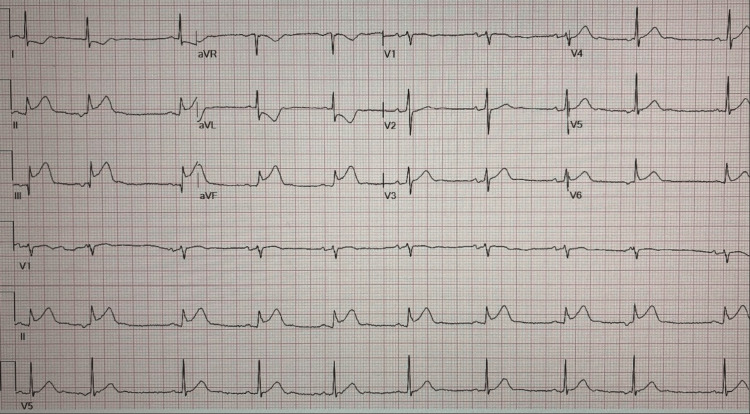
A 12-lead electrocardiogram showing ST elevation in leads II, III, and aVF

On physical exam, he had normal vital signs and a normal cardiac examination without any murmurs. His hemoglobin was 14.9 g/dl, Platelet count was 103,000 /µL, and initial troponin was negative; other laboratory investigations were non-remarkable. Chest X-ray was normal, and electrocardiogram (Figure 1) showed ST elevation in leads II, III, and aVF.

**Figure 2:**
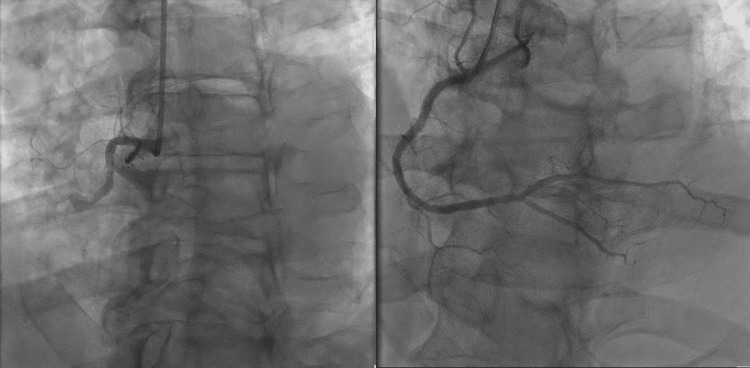
Coronary angiogram showing right coronary angiogram occlusion (left image) and post stent placement (right image)

The patient received aspirin, clopidogrel, and heparin, and emergent cardiac catheterization was performed, which showed 100% occlusion of the right coronary artery (RCA) in addition to 70% stenosis at the middle left anterior descending artery (LAD) (Figure 2). As a result, two drug-eluting stents were placed in the RCA with a planned staged procedure for the LAD. The patient was started on eptifibatide infusion and was moved to the coronary care unit in stable condition.

The following morning, the patient started having gross hematuria and a petechial rash. Laboratory work-up revealed severe thrombocytopenia with a platelet count of 3000/ µL, hemoglobin of 12.3 g/dl, normal haptoglobin level, and negative heparin-platelets antibodies. Aspirin, clopidogrel, and eptifibatide were held, and one unit of platelets was transfused, which raised his platelet count to 14000/ µL. Over the next two days, the platelet count trended up slowly to 35000/ µL, aspirin and clopidogrel were resumed, and the patient’s hematuria resolved. The patient was discharged home after 72 hours of hospitalization.

Following hospital discharge, the patient was followed closely in clinic the next week. His platelet count was 74000/ µL, and he continued aspirin and clopidogrel without bleeding issues. He reported a return to baseline activity and was now chest pain-free (Figure 2).

**Figure 3:**
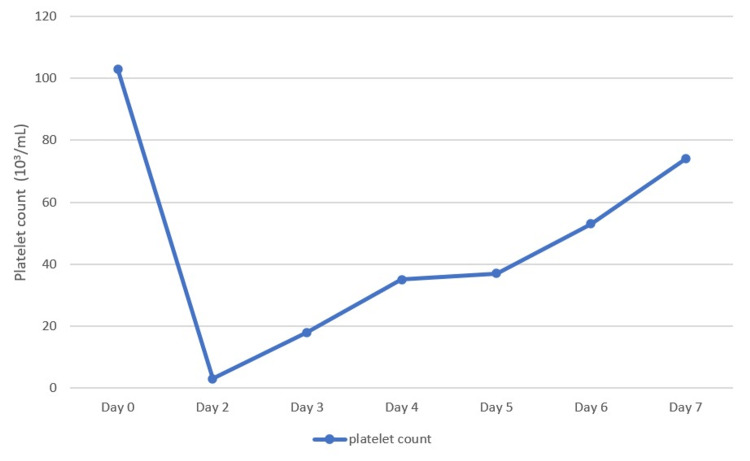
Platelet count trend during the hospital stay

## Discussion

Eptifibatide is a platelet glycoprotein (GP) IIb/IIIa inhibitor that reversibly inhibits platelet activation. It has been associated with decreased incidence of death or non-fatal myocardial infarction in patients with acute coronary syndrome but with higher bleeding risks in some studies. Other studies showed no difference in outcome; thus, its use has significantly decreased in the last decade and is currently limited to patients with high thrombus burden or no-reflow after a percutaneous coronary intervention (PCI) [2, 3]. The bolus and the following infusion inhibit platelet aggregation within a minute and with an effect lasting for four to six hours [4].

A significant but less reported side effect of eptifibatide is a profound drop in platelet count that occurs within hours after the infusion [1]. The mechanism of profound thrombocytopenia is poorly understood and is thought to be antibody-mediated, but antibody-independent pathways have also been proposed [5,6]. Most patients develop minor bleeding, including petechiae or trivial bleeding from catheter sites. However, major bleeding, such as gastrointestinal bleeding or hematuria, has been reported, and some patients developed life-threatening bleeding necessitating further management [6,7]. 

Profound thrombocytopenia is usually managed by stopping the medication with the platelet count returning to normal in one to five days. Platelets transfusion might be necessary if platelet count drops to below 10000/ µL, as well as stopping other antiplatelet therapies. However, platelet recovery can be longer and might take up to two weeks. The use of corticosteroids is limited but can be considered in severe refractory cases complicated by life-threatening bleeding, while the use of intravenous immunoglobulin (IVIG) is not recommended [8,9]. Some studies showed that those patients might be at increased risk of thrombosis due to paradoxically high platelet activity, putting them at an increased risk of acute stent thrombosis [10,11].

In our case, the patient presented with gross hematuria within 12 hours of starting the eptifibatide infusion. Eptifibatide was held, and his platelet count recovered to more than 70000 /µL within one week, but there was a necessary interruption of dual antiplatelet therapy when profound thrombocytopenia occurred.

## Conclusions

In conclusion, we described a patient who developed profound thrombocytopenia that led to hematuria which resolved spontaneously. Profound thrombocytopenia is a rare side effect of eptifibatide infusion through a poorly understood mechanism. It usually resolves within one to five days by stopping the medications. Management also includes platelet transfusion and holding antiplatelet medications until recovery. However, those measures might put the patient at a higher risk of stent thrombosis. More studies are needed to support the management of those patients with refractory thrombocytopenia.
